# A facile synthetic strategy for iron, aniline-based non-precious metal catalysts for polymer electrolyte membrane fuel cells

**DOI:** 10.1038/s41598-017-05830-y

**Published:** 2017-07-14

**Authors:** Hyunjoon Lee, Min Jeong Kim, Taeho Lim, Yung-Eun Sung, Hyun-Jong Kim, Ho-Nyun Lee, Oh Joong Kwon, Yong-Hun Cho

**Affiliations:** 1 Center for Nanoparticle Research, Institute for Basic Science (IBS), Seoul, 08826 Republic of Korea; 2 School of Chemical and Biological Engineering, Seoul National University (SNU), Seoul, 08826 Republic of Korea; 3 Department of Chemical Engineering, Soongsil University, 369 Sangdo-ro, Dongjak-gu, Seoul 06978 Republic of Korea; 4 Surface Technology Center, Korea Institute of Industrial Technology (KITECH), 7-47, Songdo-dong, Incheon, 406-840 Republic of Korea; 5 Department of Energy and Chemical Engineering, Incheon National University, 12-1, Songdo-dong, Yeonsu-gu, Incheon 22012 Republic of Korea; 6 Department of Chemical Engineering, Kangwon National University, Samcheok, Kangwon-do 25913 Republic of Korea

## Abstract

The development of a low cost and highly active alternative to the commercial Pt/C catalysts used in the oxygen reduction reaction (ORR) requires a facile and environmentally-friendly synthesis process to facilitate large-scale production and provide an effective replacement. Transition metals, in conjunction with nitrogen-doped carbon, are among the most promising substitute catalysts because of their high activity, inexpensive composition, and high carbon monoxide tolerance. We prepared a polyaniline-derived Fe-N-C catalyst for oxygen reduction using a facile one-pot process with no additional reagents. This process was carried out by ultrasonicating a mixture containing an iron precursor, an aniline monomer, and carbon black. The half-wave potential of the synthesized Fe-N-C catalyst for the ORR was only 10 mV less than that of a commercial Pt/C catalyst. The optimized Fe-N-C catalyst showed outstanding performance in a practical anion exchange membrane fuel cell (AEMFC), suggesting its potential as an alternative to commercial Pt/C catalysts for the ORR.

## Introduction

Electrocatalysts for the oxygen reduction and evolution reactions are considered crucial for the development of sustainable energy storage and conversion devices, such as metal-air batteries, water splitting processes, and fuel cells^1–4^. Oxygen reduction electrocatalysts are especially important in polymer electrolyte fuel cells (PEMFCs) because the oxygen reduction reaction (ORR) catalysts are a key factor in the performance and cost of the fuel cells. The use of a highly active and inexpensive ORR catalyst is vital for the widespread introduction of fuel cell-powered systems. Platinum (Pt) is the most prevalent and active catalyst for ORR in both acidic and basic electrolytes. However, the high cost of Pt is one of the barriers to the commercialization of fuel cell technology. Since the cost of the catalyst layer is responsible for ~46% of the total material costs of a fuel cell stack, many researchers have focused on decreasing the Pt loading by modifying the surface structure and composition of the catalyst (e.g., using Pt alloys, core-shell structures, and nanostructured thin films) to reduce the cost^5–8^. However, the scarcity and low CO tolerance of Pt has led to many attempts to develop Pt-free catalysts from non-Pt group metals (non-PGM) to replace the Pt-based systems. These non-platinum catalysts not only solve the problems caused by Pt metal but also dramatically decrease the cost of the catalyst, and new non-PGM systems with high ORR activity are now sought.

Carbon-based non-PGM catalysts, particularly N-doped carbon catalysts in conjunction with transition metals (TM-N-C), have been studied extensively^9–11^. These catalysts are currently considered to be the most promising candidates to replace Pt-based systems because of their high ORR activity and CO tolerance and inexpensive materials. The first use of a TM-N-C-based ORR catalyst was reported in 1964 by Jasinski^12^, who used a Co phthalocyanine. However, metal-containing macrocycles suffer from poor stability in acidic electrolytes and expensive precursor materials. Thus, numerous alternative N- and C-containing precursors have been suggested for the synthesis of TM-N-C^13, 14^. The use of N-containing polymers such as polyaniline (PANI), melamine resin, polypyrrole, and dopamine as a source of both N and C was shown to be more facile than the conventional N-doping method, which includes heat treatment in an atmosphere of gaseous ammonia^15–20^. Nitrogen-containing polymers also enable N-doped sites to be distributed homogeneously. PANI-derived TM-N-C catalysts have frequently been reported as ORR catalysts in both alkaline and acidic electrolytes. Wu *et al*. reported a PANI-derived Fe-Co-doped carbon catalyst that exhibited outstanding performance and durability in an acidic electrolyte^19^. Li *et al*. synthesized a Fe-N-CNT catalyst with a half-wave potential that was 30 mV more positive than Pt/C catalysts in alkaline electrolytes^21^. Currently, the low material costs of PANI-based catalysts, compared to Pt-based catalysts, are outweighed by the high manufacturing costs arising from the complicated and time-consuming processes for production of PANI-based catalysts. For example, the synthesis process reported by Ferrandon *et al*.^22^ and Zamani *et al*.^23^ takes longer than 24 h even when heat treatment steps are excluded.

**Figure 1:**
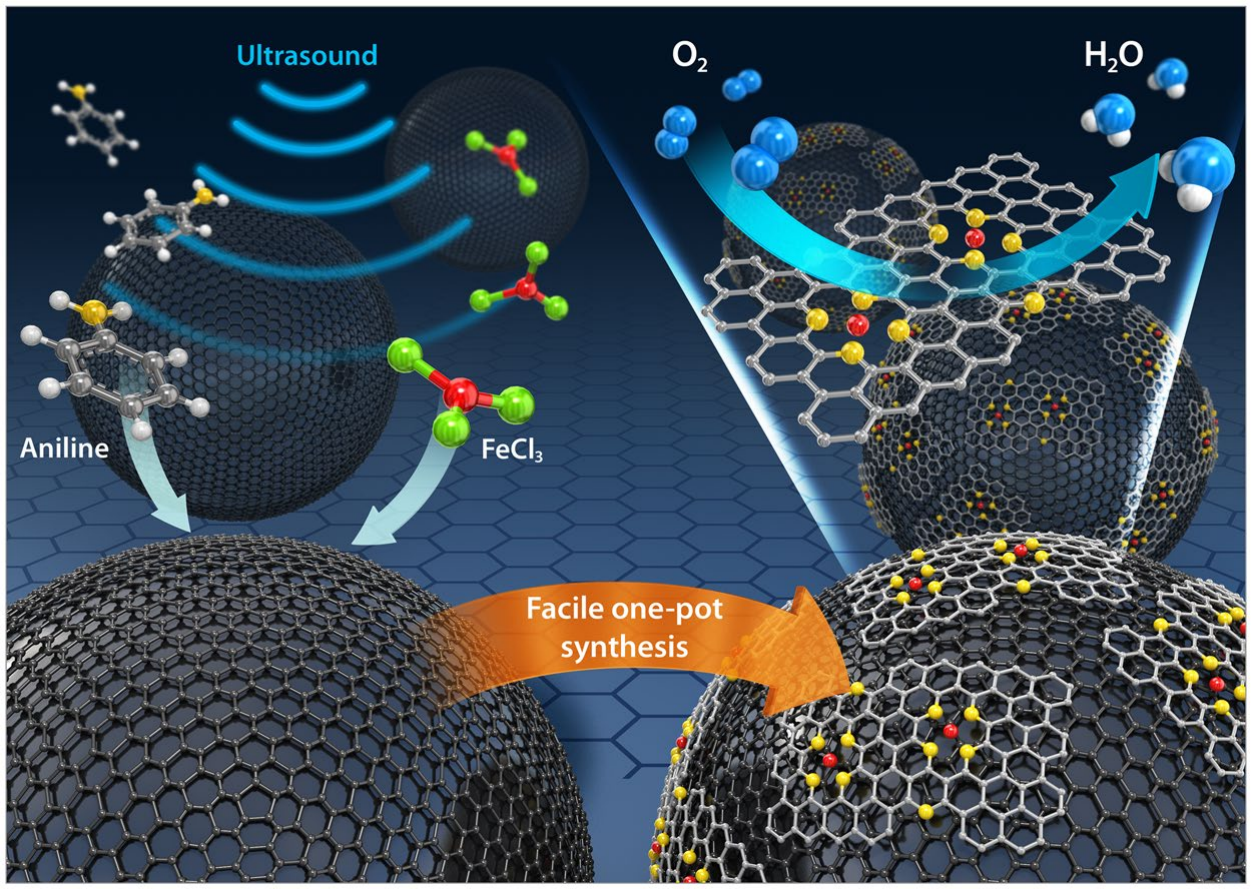
Conceptual diagram of our facile method.

Herein, we developed an effective synthesis of a PANI-derived Fe-N-C catalyst by a one-pot method that minimized the requirement for several of the noxious components used previously, including oxidants, reductants, and acidic solvent (e.g., ammonium persulfate, (APS), sodium borohydride, and HCl, respectively). The omission of APS, which is the second highest-priced component among the materials, could further reduce the total manufacturing cost. In this study, the aniline, Fe precursor, and carbon support were simply mixed under ultrasound irradiation in one pot for direct reaction. It is supposed that ultrasound plays two kinds of roles. It could affect the crystallinity and structure of polyaniline which might have something to do with the active sites after a pyrolysis^24^. In addition, it is able to reduce the polymerization time and cause an abrupt nucleation throughout the solution^25^, which can help forming uniform and homogeneous distribution of active sites for ORR. The conceptual diagram of our facile method is showed in Fig. 1. The Fe-N-C catalysts exhibited an ORR catalytic activity comparable to that of commercial Pt/C catalysts. Furthermore, we evaluated the practical single-cell performance of a membrane-electrode assembly (MEA) that employed the synthesized Fe-N-C as the cathode.

## Results and Discussion

### Optimization of pyrolysis temperature

We studied the electrocatalytic activity of Fe-pyPANI-K catalysts, in which the notation “py” denotes that the PANI was pyrolyzed at the temperature specified at the end of the sample name and “-K” denotes a sample that contains carbon black. The Fe-PANI-K catalysts were synthesized by simultaneously mixing the metal precursor, aniline monomer and carbon black (AkzoNobel, Ketjen Black EC-300J) while irradiating ultrasound. The effect of ultrasound irradiation on the catalyst synthesis was clarified by synthesizing Fe-pyPANI-K 700 °C with and without ultrasound irradiation. An element composition of both Fe-PANI-Ks was same (C, O, Fe, N and Cl). However, the iron contents and catalytic activity were different form each other (Fig. S1). From the results, we could infer that the ultrasound irradiation increases the iron contents in Fe-PANI-K and it enhances the catalytic activity.

**Figure 2:**
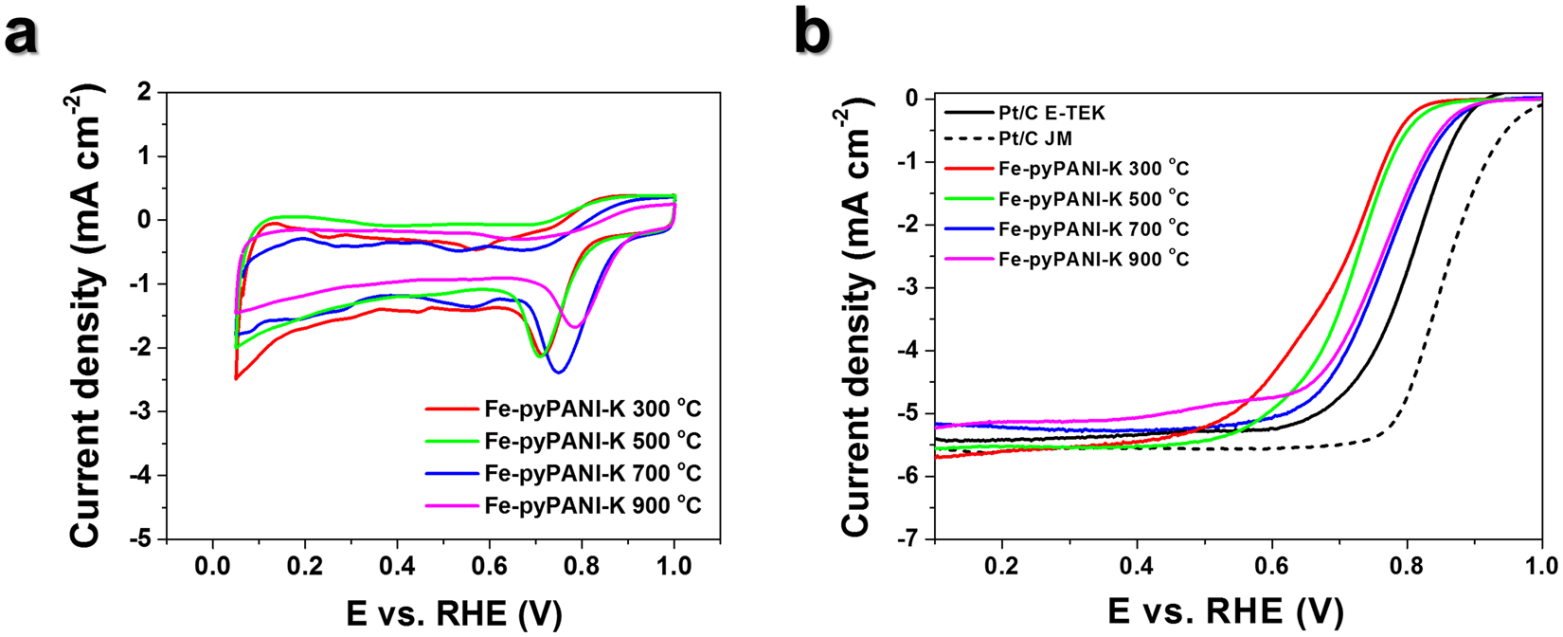
Electrochemical characterization for Fe-PANI-Ks pyrolyzed at difference temperature. (**a**) CV curves (**b**) ORR performance curves on RDE at 1600 rpm. Both CV and LSV data were obtained in O_2_ saturated 0.1 M KOH solutions at 25 °C and catalyst loading for Pt/C and Fe-pyPANI-Ks was 0.24 mg cm^−2^.

Cyclic voltammetry (CV) and ORR activity results for samples that were pyrolyzed at different temperatures are shown in Fig. 2a and b. The CV data in Fig. 2a shows that the oxidation peak current densities between 0.6–0.8 V increased at 700 °C but significantly decreased at 900 °C. The peak positions shifted to a more positive potential as the pyrolysis temperature was raised. Figure 2b more clearly describes the characteristic of each catalyst. The on-set potential and half-wave potential shifted to positive potential from 300 °C to 700 °C, while the Fe-pyPANI-K 900 °C was slightly reduced compared to Fe-pyPANI-K 700 °C. The decreased ORR performance at 900 °C might be attributed to a reduction of active site in catalyst. Since Fe is strongly associated with the active sites by its contribution to active site itself or formation of active site^26–29^, the reduced active sites in catalyst was ascribed to the aggregation of Fe particles. The Fe particles aggregated as the pyrolysis temperature increased, as shown in Fig. S2.

**Figure 3:**
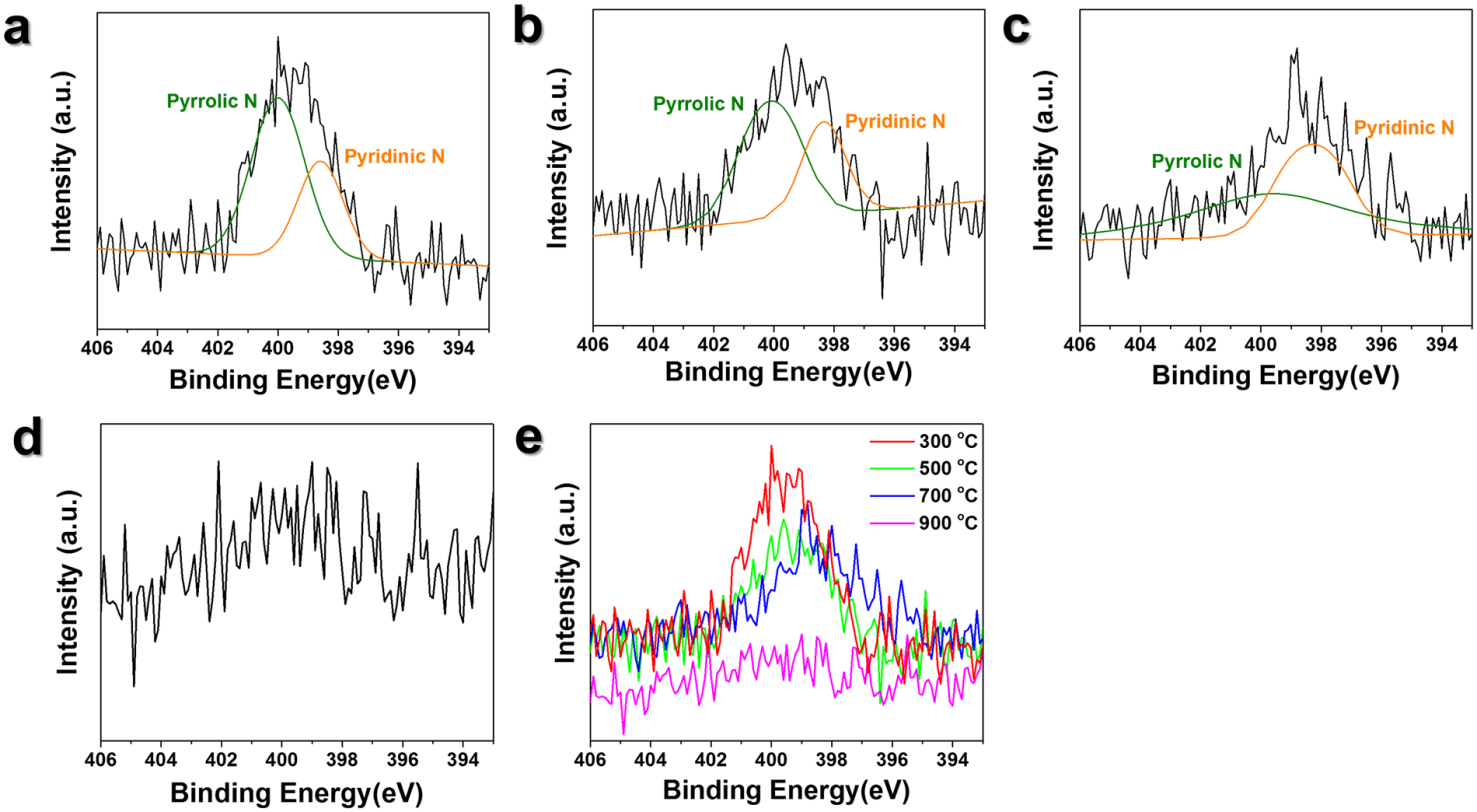
XPS analysis for N1s of Fe-pyPANI-K pyrolyzed at various temperatures. (**a**) 300 °C, (**b**) 500 °C, (**c**) 700 °C, (**d**) 900 °C (**e**) variation of XPS peak shift with pyrolysis temperature. (**a**–**c**) Green line indicates pyrrolic N and Orange line indicates pyridinic N.

The shift observed in the X-ray photoelectron spectroscopy (XPS) data shown in Fig. 3 explains the onset potential shift shown in Fig. 2b. We suggest that an N-metal or pyridinic-N species is responsible for the catalytic activity of non-precious metals and nitrogen-doped carbon materials^30^. As shown in Fig. 3e, the overall N1s peak shifts to a lower binding energy as the pyrolysis temperature was increased from 300 °C to 700 °C, indicating that pyrrolic-N species are transformed into pyridinic-N species. Pyrrolic-N atoms have a specific peak at ~400.1 eV while the characteristic peak of pyridinic-N is located at ~398.2 eV^27^. The ratios of pyrrolic-N to pyridinic-N were 1.63, 1.28, and 0.47 for pyrolysis temperatures of 300, 500, and 700 °C, respectively. XPS peak was not clearly observed for nitrogen at 900 °C. The ratio of pyrrolic N to pyridinic N of Fe-pyPANI-K at 300 °C and 500 °C is not changed significantly and it is more clearly verified from the peak overlap shown in Fig. 3e. The peak current of the samples pyrolyzed at 300 °C and 500 °C have same position in Fig. 2a and similar on-set potential in Fig. 2b. This means that the characteristic of active sites is similar, and it is supported by XPS data. In spite of same peak position and similar on-set potential, the half-wave potential at 300 °C is slightly lower than that at 500 °C, because Fe-PANI-K is not perfectly pyrolyzed thus active site was partially exposed to the reactants. As shown in Fig. S3, an as-prepared Fe-PANI-K sample is almost completely decomposed at ~500 °C. Only oligomers and low molecular weight polyaniline species decompose at 300 °C. Thus, the active sites form at 300 °C may be partially covered with polyaniline. Positive shifts in the peak position and onset potential were observed when the pyrolysis temperature exceeded 700 °C, which is attributed to the associated increase in pyridinic-N and the decrease in pyrrolic-N shown in Fig. 3e. There was no distinct change in onset potential at 900 °C. However, the half-wave potential and limiting current density were slightly reduced, as would be expected from the XPS data shown in Fig. 3d. The activity degradation at higher temperature was caused by the decreased nitrogen content. Mo *et al*. also reported that decreasing nitrogen content could be a cause of activity loss in a Fe-based catalyst^31^. Thus, the loss of active sites at 900 °C was caused by the aggregation of Fe particles and the reduced nitrogen content.

### Post-treatment of Fe-pyPANI-K

**Figure 4:**
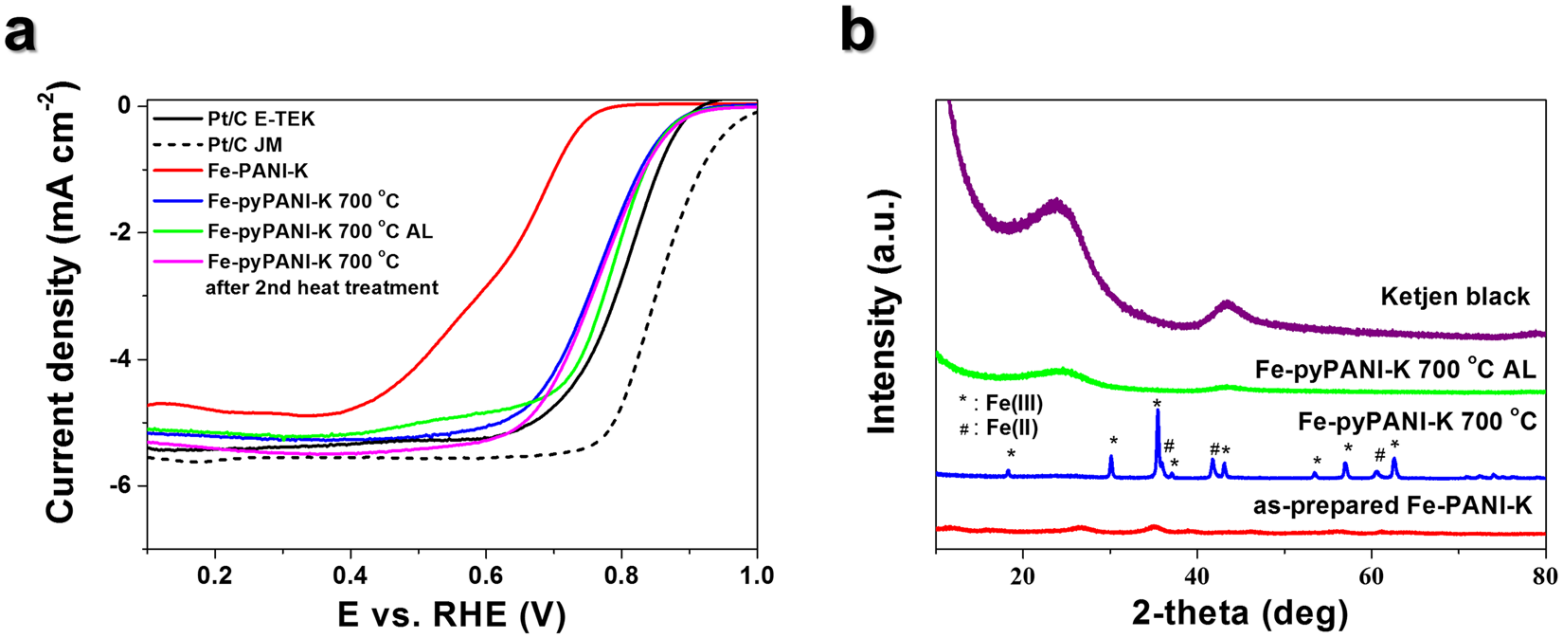
Electrochemical characterization and XRD analysis. (**a**) ORR performance graphs for the different types of catalyst on 0.24 mg cm^−2^ catalyst loaded RDE at 1600 rpm in 25 °C O_2_ saturated 0.1 M KOH electrolyte, (**b**) XRD patterns obtained for different catalyst types.

Post-treatment is common for Fe-based catalysts synthesized using aniline as a nitrogen source. The post-treatment usually consists of three steps: a heat treatment to carbonize aniline; acid leaching (AL) to remove impurities and iron oxide; and a second heat treatment to graphitize any remaining carbon^32^. Figure 4a shows the linear sweep voltammetry (LSV) graphs for ORRs conducted with as-prepared and post-treated catalysts. The LSV half-wave potentials showed dramatic positive shifts of 130 mV between Fe-PANI-K and Fe-pyPANI-K 700 °C, and 20 mV between Fe-pyPANI-K 700 °C and Fe-pyPANI-K 700 °C AL. The Fe-pyPANI-K 700 °C showed an improved catalytic activity over Fe-PANI-K because pyrolysis leads to the aggregation of Fe ions and the formation of Fe oxides, as shown in Fig. 4b. Fe oxides contribute to the formation of active sites. The detailed XRDs of as-prepared Fe-PANI-K and Fe-pyPANI-K 700 °C AL were shown in Fig. S4. Many unidentified peaks of as-prepared Fe-PANI-K comes from polyaniline or oligoaniline^24^.

Acid leaching caused a positive shift in the half-wave potential of the ORR for Fe-pyPANI-K 700 °C, and the effectiveness of AL was clearly shown by the XRD patterns. The reflections in the XRD patterns (Fig. S4) corresponding to Fe oxide disappeared after acid leaching, and the dissolution of Fe oxide was also observed in the field-emission scanning electron microscopy (FE-SEM) images shown in Fig. S5a,b. The dissolution of Fe oxide made more active sites exposed to the reactant and boosting the activity in the Fe-pyPANI-K 700 °C catalyst. In BET analysis results (Fig. S6), BET surface area of Fe-pyPANI-K 700 °C AL was larger than Fe-pyPANI-K 700 °C. This result indicated that Fe oxide was blocking the surface of the catalyst.

**Figure 5:**
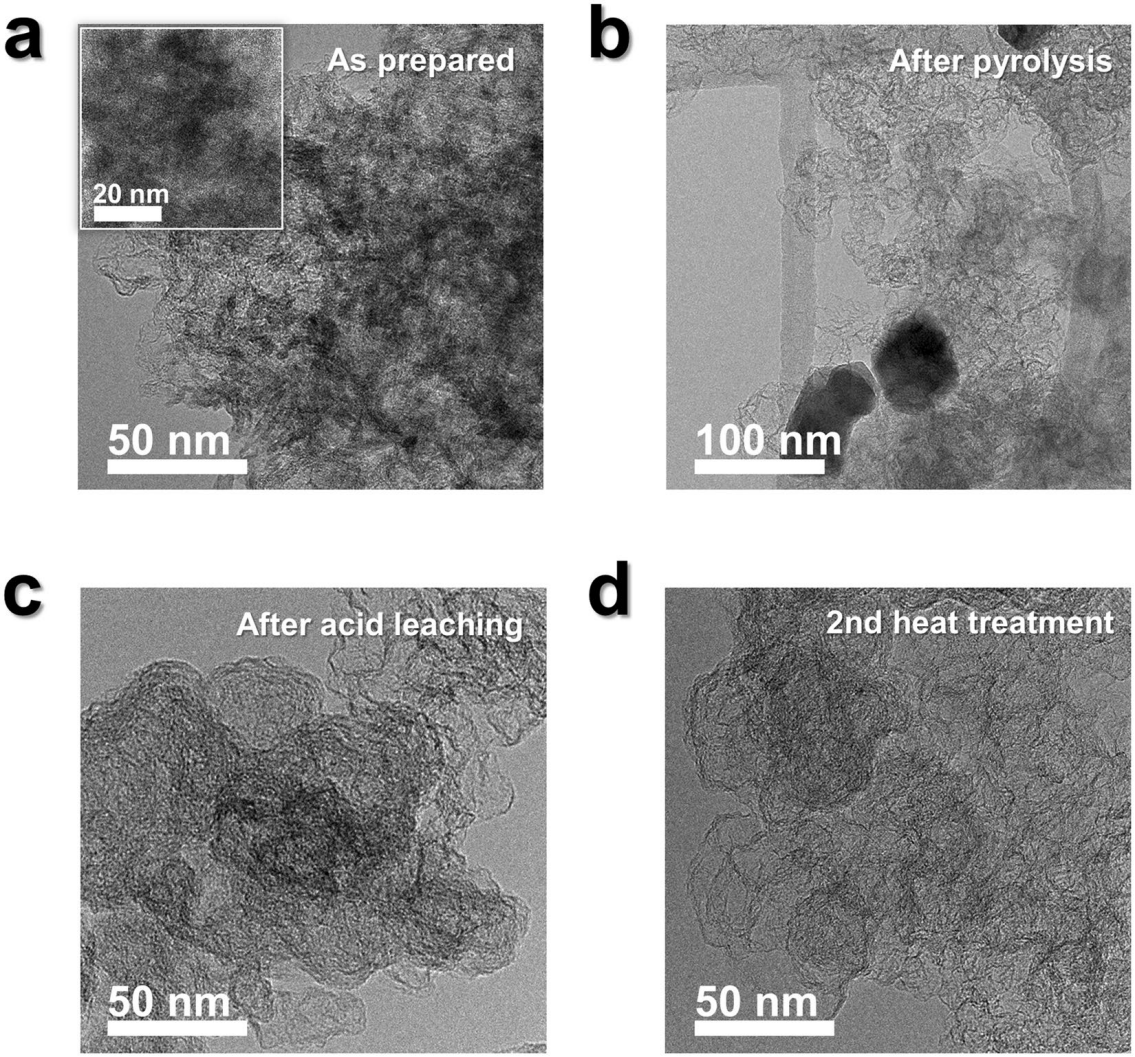
TEM analysis of the different catalyst types. (**a**) Fe-PANI-K, (**b**) Fe-pyPANI-K 700 °C, (**c**) Fe-pyPANI-K 700 °C AL, (**d**) Fe-pyPANI-K 700 °C AL after the second heat treatment. Inset image in (**a**) is more magnification image of as prepared Fe-PANI-K and scale bar is 20 nm.

Morphological changes in the catalysts were investigated by transmission electron microscopy (TEM), as shown in Fig. 5. While the Fe is uniformly distributed over the carbon black in Fe-PANI-K (Fig. 5a), pyrolysis causes aggregation of the Fe, as shown in Fig. 5b. The aggregations were composed of Fe oxide and were hundreds of nanometers in size. The presence of Fe oxide was confirmed using both XRD (Fig. 4b) and TEM-energy dispersive X-ray spectroscopy (EDS) (Fig. S7). However, Fe oxide was not observed in the XRD (Fig. 4b) and TEM data (Fig. 5c) after acid leaching, confirming its complete removal. These data also showed that the graphitic structure of carbon black was disrupted after acid leaching since no reflections corresponding to the graphitic structure of Ketjen black were be found in the XRD pattern after acid leaching.

Previous reports have concluded that the degree of graphitization induced by heat treatment is linked to the catalytic activity^30^, and that a high degree of graphitization is desirable for high activity. However, the heat treatment is reportedly also cause excessive loss of surface area. We found that the half-wave potential moved to a more negative potential after second heat treatment at 900 °C; a result that contrasts with previous reports^30, 33^. We suggest that this contrast indicates that graphitization did not occur during the second heat treatment in this study; indeed the TEM images (Fig. 5c and d) show no difference in the catalyst’s appearance before and after the second heat treatment. The TEM image in Fig. 5d shows the same structure as that of Ketjen black, shown in Fig. S8, which has a graphitic structure (Fig. 4b). Furthermore, the Raman analysis in Fig. S9 indicated that the ratio of the G and D bands did not increase for the Ketjen black after the second heat treatment, suggesting that carbon black was not further graphitized.

### Anion exchange membrane fuel cells

**Figure 6:**
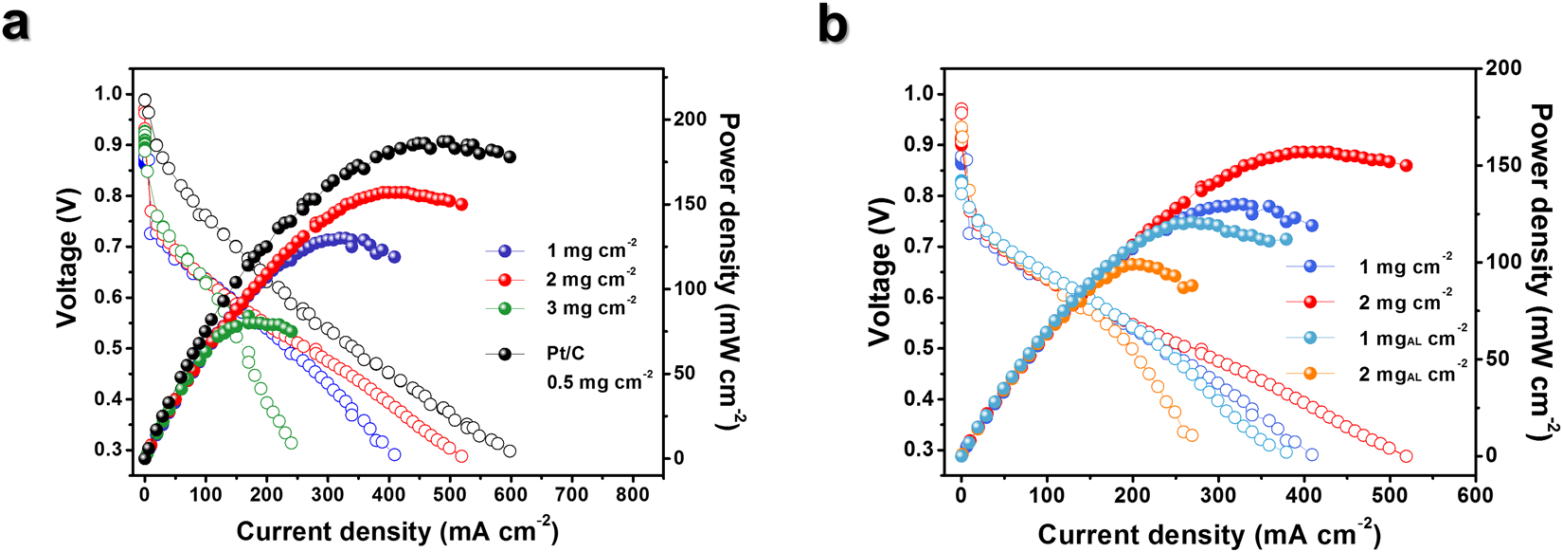
AEMFC performance evaluation. (**a**) I–V curves of AEMFCs containing Fe-pyPANI-K 700 °C and Pt/C cathodes. The loading of the Fe-pyPANI-K 700 °C catalyst was changed from 1 mg cm^−2^ to 3 mg cm^−2^, and it was 0.5 mg cm^−2^ for Pt/C (**b**) I–V curves of AEMFCs with Fe-pyPANI-K 700 °C and acid leached-Fe-pyPANI-K 700 °C cathodes with loadings of 1 mg cm^−2^ and 2 mg cm^−2^.

The catalytic performance of the Fe-pyPANI-K 700 °C catalyst was evaluated for the ORR in an H_2_/O_2_ anion exchange membrane fuel cell (AEMFC) at 50 °C for catalyst loadings of 1–3 mg cm^−2^ (Fig. 6a). The Fe-pyPANI-K 700 °C AL catalyst was also tested under the same measurement conditions (Fig. 6b) but with loadings of 1 and 2 mg cm^−2^ to find the effect of acid leaching on the performance of a membrane-electrode assembly (MEA). The optimum catalyst loading for Fe-pyPANI-K 700 °C was found to be 2 mg cm^−2^. The current density at 0.6 V was 139 mA cm^−2^ and maximum power density of Fe-pyPANI-K 700 °C was 157 mW cm^−2^, which was 83% of that of an MEA using commercial Pt/C as cathode catalyst. The performance of the catalyst deteriorated at a loading of 3 mg cm^−2^ because of high ohmic and mass transfer resistance. The increased catalyst loading caused the catalyst layer thickness to increase from 30 μm to 96 μm (Fig. S10), which hindered the transport of the reductant, product and hydroxide ions^34^. Single cell MEAs exhibited lower performance for both 1 mg cm^−2^ and 2 mg cm^−2^ catalyst loadings after the acid leaching treatment loaded, in contrast to that seen for the half-cell test results. The difference between the two MEAs could be confirmed in a high current density region where the cell voltage loss was dominated by mass transfer^34, 35^. We interpret these results to imply that the iron oxide acts as a pore former and facilitates the transport of the reactant and product through the catalyst layer, as evident from the decreased performance of the higher catalyst loading after the acid leaching process.

## Conclusion

We have demonstrated a facile synthesis strategy for iron- and aniline-based non-precious metal catalysts for the oxygen reduction reaction. Our facile method produced a Fe-PANI-K catalyst by mixing and sonicating without any chemical additives and solvent. We found that 700 °C was an optimum pyrolysis temperature based on electrochemical analysis, FE-SEM, and XPS results that showed that the quality of the ORR active sites was poor at low pyrolysis temperatures and that the ORR active site density was reduced at high temperature. The as-synthesized Fe-pyPANI-K 700 °C was post-treated to improve the ORR performance. The acid-leached catalyst exhibited the best performance because a greater number active sites were exposed to the reactants. The half-wave potential of the acid leached catalyst was only 10 mV more negative than that of conventional Pt/C. Finally, the performance of a Fe-pyPANI-K 700 °C AL-based MEA was measured in an AEMFC and feasibility could be verified. Consequently, our facile synthesis method has provided a new method to make non-precious metal catalysts for the ORR.

## Methods

### Synthesis of Fe-PANI-K

Iron(III) chloride hexahydrate (FeCl_6_·6H_2_O) (0.5 g), aniline monomer solution (10 mL, Sigma-Aldrich) and carbon black (0.1 g, AkzoNobel, Ketjen black EC-300J) were mixed together with vigorous stirring for 30 min and then purged with argon for an additional 30 min. The mixture was treated with ultrasound for 3 h at 10 °C using an ultrasonic generator (Branson, 20 kHz, 100 W cm^−2^). After ultrasonic treatment, the mixture was left overnight at room temperature. The Fe-PANI-K was separated from the mixture by centrifugation for 30 min (Labogene, 1236 mg). The isolated Fe-PANI-K was dispersed in ethanol (Daejung, 94.5%) and filtered through filter paper (membrane filter, ADVANTEC, cellulose acetate 0.45 µm) to remove any residual aniline and iron precursors. The filtered Fe-PANI-K was perfectly dried in air.

### Post-treatment of Fe-PANI-K

The post-treatment process comprised three steps; pyrolysis, acid leaching, and a second heat treatment. The pyrolysis step was conducted by heat-treating Fe-PANI-K in a tube furnace (Dae Heung Science, DTF-60600-PTFV) under a nitrogen atmosphere at various temperatures for 2 h. The temperatures used were 300 °C, 500 °C, 700 °C and 900 °C. Acid leaching of Fe-pyPANI-K 700 °C was used to remove impurities and iron oxide from the samples. The samples were treated in H_2_SO_4_ (0.5 M) for 8 h at 80 °C. After acid leaching, the samples were thoroughly washed with de-ionized water. The second heat treatment was intended to recrystallize the carbon black; samples were heated in a tube furnace at 900 °C under a nitrogen atmosphere for 3 h.

### Electrochemical analysis

Cyclic voltammetry and linear sweep voltammetry were carried out using a rotating disk electrode (RDE) measurement. They were conducted with a potentiostat (Princeton Applied Research, PARSTAT 2273) using a three-electrode electrochemical cell. A glassy carbon electrode (Pine, 0.196 cm^2^) was used as a working electrode, and a Pt coil was used as a counter electrode. The potential was reported with reference to an Ag/AgCl electrode. The catalyst ink was prepared by dispersing the synthesized catalysts (10 mg) in a mixture of isopropyl alcohol (1000 µL, JUNSEI) and Nafion ionomer (66 µL of a 5 wt.% stock solution, DuPont). The ink was treated with ultrasound for 30 min. The uniformly dispersed catalyst ink (5 µL) was loaded onto the glassy carbon working electrode. Pt/C catalyst (20 wt.%, E-TEK) or Pt/C catalyst (20 wt.%, Johnson Matthey Co., HiSPEC 3000) were also loaded on the glassy carbon electrode using the same preparation process as for the synthesized catalyst to compare the CV and LSV results of Pt/C with those of the synthesized catalyst. CV curves were recorded in a nitrogen-saturated KOH solution (0.1 M) at a scan rate of 50 mV s^−1^. The potential was scanned between 0.05 V and 1.0 V vs. RHE. For LSV curves, the RDE was rotated at 1600 rpm in an O_2_-saturated KOH solution (0.1 M), and the potential was varied at a scan rate of 5 mV s^−1^. For all electrochemical analysis, the solutions were kept at 25 °C using a thermostatic bath.

### Anion exchange membrane fuel cell test

A Pt/C catalyst (40 wt.%, Johnson Matthey Co.) served as the anode for all AEMFC tests in this work. Fe-pyPANI-K 700 °C and acid leached-Fe-pyPANI-K 700 °C served as the cathode. Each catalyst was dispersed in a mixture of isopropyl alcohol, deionized water, and ionomer (AS-4, Tokuyama) to prepare the catalyst ink. The ink was ultrasonicated and sprayed onto an anion exchange membrane (A901, Tokuyama). The catalyst-coated membrane was dried for 1 day before its assembly into a single cell. The volume of catalyst ink was controlled to deposit 0.5 mg cm^−2^ on the anode and 1, 2, or 3 mg cm^−2^ on the cathode. For reference, Pt/C (20 wt.%, Johnson Matthey Co.) was used as cathode catalyst with a loading of 0.5 mg cm^−2^. Carbon paper containing microporous layers (MPLs; 35BC, SGL) was used as a gas diffusion layer (GDL), which was placed on both the cathode and anode sides of the membrane. The MEAs were inserted into a single-cell unit that had a graphite plate with a serpentine gas flow channel (5 cm^2^ geometric area). A single-cell unit was assembled with eight screws and a tightening torque of 8 Nm.

The performance of the assembled single cell was evaluated using the current sweep method with a Fuel Cell Test System (CNL energy Co., Korea). Before each single cell test, both the anode and cathode were fed with fully humidified H_2_ gas and O_2_ gas, respectively, at a constant flow rate. The temperature of the single cell was maintained at 50 °C during the measurements. When the open circuit voltage was stabilized, the polarization curves of the single cell were measured from open circuit voltage to 0.3 V. The current was reset to zero when the cell voltage reached 0.30 V. The total outlet pressure was 150 kPa.

## Electronic supplementary material

Supplementary Information
