# Supplementary material for: A facile synthetic strategy for iron, aniline-based non-precious metal catalysts for polymer electrolyte membrane fuel cells
Source: Sci Rep. 2017 Jul 14;7:5396. doi: 10.1038/s41598-017-05830-y (PMC5511131; doi:10.1038/s41598-017-05830-y)
Supplement: Supplementary file 1 — Supplementary Information [file 41598_2017_5830_MOESM1_ESM.doc]

**Supplementary Information**

**A facile synthetic strategy for iron, aniline-based non-precious metal catalysts for polymer electrolyte membrane fuel cells**

Hyunjoon Lee^1,2†^, Min Jeong Kim^1,2†^, Taeho Lim^3^, Yung-Eun Sung^1,2^, Hyun-Jong Kim^4^, Ho-Nyun Lee^4^, Oh Joong Kwon^5,*^ and Yong-Hun Cho^6,*^

^1^Center for Nanoparticle Research, Institute for Basic Science (IBS), Seoul 08826, Republic of Korea

^2^School of Chemical and Biological Engineering, Seoul National University (SNU), Seoul 08826, Republic of Korea

^3^ Department of Chemical Engineering, Soongsil University, 369 Sangdo-ro, Dongjak-gu, Seoul 06978, Republic of Korea

^4^ Surface Technology Center, Korea Institute of Industrial Technology (KITECH), 7-47, Songdo-dong, Incheon 406-840, Republic of Korea

^5^Department of Energy and Chemical Engineering, Incheon National University, 12-1, Songdo-dong, Yeonsu-gu, Incheon 22012, Republic of Korea

^6^ Department of Chemical Engineering, Kangwon National University, Samcheok, Kangwon-do 25913, Republic of Korea

Correspondence and requests for materials should be addressed to O. J. K. (E-mail: ojkwon@inu.ac.kr) or to. Y.-H. C. (E-mail: yhun00@kangwon.ac.kr)

^†^ These authors contributed equally to this work.

**Explanation of Supplementary Figure**

**Figure S1.**

XPS analysis was performed as shown in Fig. S1a to confirm the difference in the composition of Fe-PANI-K synthesized with and without ultrasound irradiation. The most notable difference in XPS results is iron contents. The iron contents is higher at the Fe-PANI-K synthesized with ultrasound irradiation. It showed an enhanced catalytic activity as shown in Fig. S1b. From the results, we could infer that the ultrasound irradiation increase the iron contents in Fe-PANI-K and it enhances the catalytic activity of the Fe-PANI-K.

**Figure S2.**

Iron oxide evenly distributed over the catalyst layer before pyrolyzing the sample and after pyrolyzing at 300 °C, as it can be seen in Fig. S2a and b. As the pyrolyzing temperature was raised, an aggregation of Iron oxide happened thus the particle size of Iron oxide became large. However, the particles were still evenly distributed over the catalyst layer as shown in Fig. S2c, d and e.

**Figure S3.**

Aniline monomer almost decomposes at the temperature lower than 200 °C. Polyaniline (polyaniline emeraldine salt with the MW 15,000) showed three distinct regions while it was pyrolyzed. Water evaporates around 100 °C, small fragment such as oligomer was decomposed near 300 °C, and polyaniline is pyrolyzed between 300 °C and 750 °C. From the graph, it could be inferred that there exists small amount of water in as-prepared Fe-PANI-K which was incorporate while rinsing. As-prepared Fe-PANI-K might consist of oligoaniline and polyaniline which has shorter length than polyaniline emeraldine salt (MW 15,000) because it does show abrupt change in weight around 500 °C at which lower temperature at that of polyaniline emeraldine salt. The leftover might be iron oxide in as-prepared Fe-PANI-K. After pyrolysis at 700 °C and acid leaching, we can know that most of iron oxide disappears during acid leaching.

**Figure S4.**

The more detailed XRD patterns of as-prepared Fe-PANI-K and Fe-PANI-K 700 °C AL were shown in Fig. S4. In Fig. 4b, the XRD patterns of as-prepared Fe-PANI-K and Fe-PANI-K 700 °C AL were not well presented because peak intensities of as-prepared Fe-PANI-K and Fe-PANI-K 700 °C AL were low.

**Figure S5.**

The morphology difference between Fe-pyPANI-K 700 °C and post treated Fe-pyPANI-K 700 °C was confirmed using FE-SEM analysis. It was confirmed that iron oxide particle was perfectly disappeared in acid leached Fe-pyPANI-K 700 °C. On the other hand, in FE-SEM analysis, there was no change between acid leached catalyst and second heat treated catalyst.

**Figure S6.**

BET analysis was carried out to identify changes in BET surface area before and after acid leaching. BET surface area of the Fe-pyPANI-K 700 °C AL (1219.3 m^2^ g^-1^) was twice as large as that of the Fe-pyPANI-K 700 °C (569.53 m^2^ g^-1^). This result indicated that Iron oxide was blocking the surface of the catalyst and it might mask the active site of the catalyst.

**Figure S7.**

Iron and oxygen are clearly seen in TEM-EDS analysis. The position of iron and oxygen overlap each other, thus it could be inferred that the iron exists as iron oxide. After acid leaching, both of iron and oxygen disappear. By considering the catalyst activity of Fe-pyPANI-K 700 °C AL, this supports that iron does not involve catalyst reaction but helps the formation of active sites.

**Figure S8.**

In this study, the Ketjen black EC-300J was used in carbon support material. The TEM image showed that the Ketjen black have graphitic structure.

**Figure S9.**

Raman analysis was carried out to investigate the degree of graphitization after second heat treatment. We could know that second heat treatment is not effective in performing graphitization of pyrolyzed polyaniline in this study. There was no big difference in G/D ratio in Raman spectra after second heat treatment.

**Figure S10.**

FE-SEM cross sectional images showed that the catalyst thickness was changed according to catalyst loading amount. As the loading amount of the catalyst increases, the thickness of catalyst layer became thicker.

**Supplementary Figures**


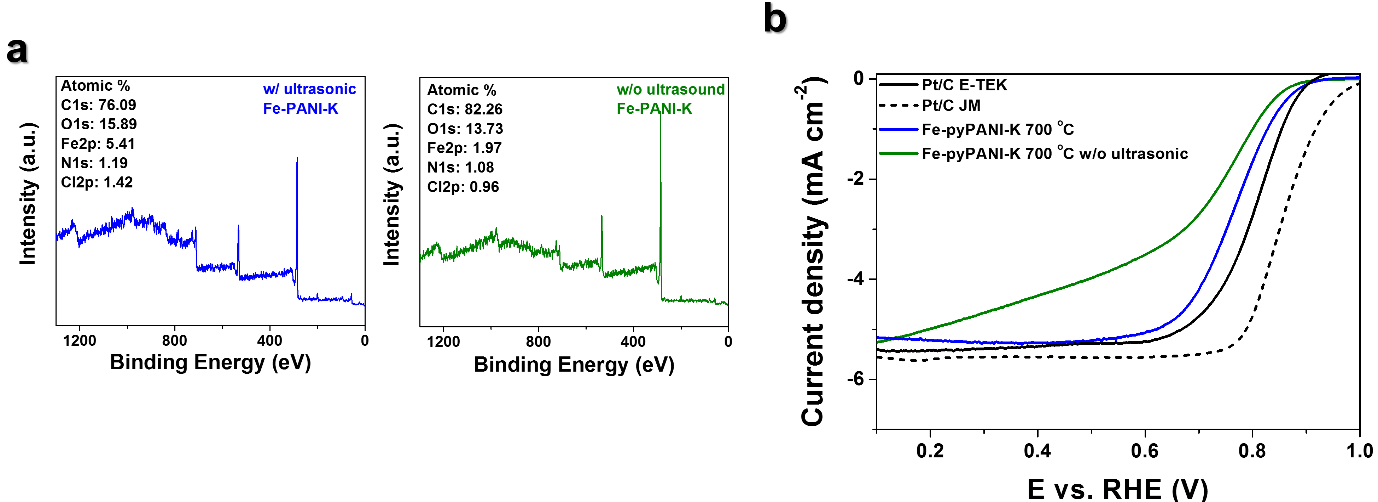


**Figure S1.** (**a**) XPS spectra of Fe-PANI-K syntehsized with and without ultrasound irradiation (**b**) ORR graphs for Fe-pyPANI-K 700 °C synthesized with and without ultrasound irradiation.


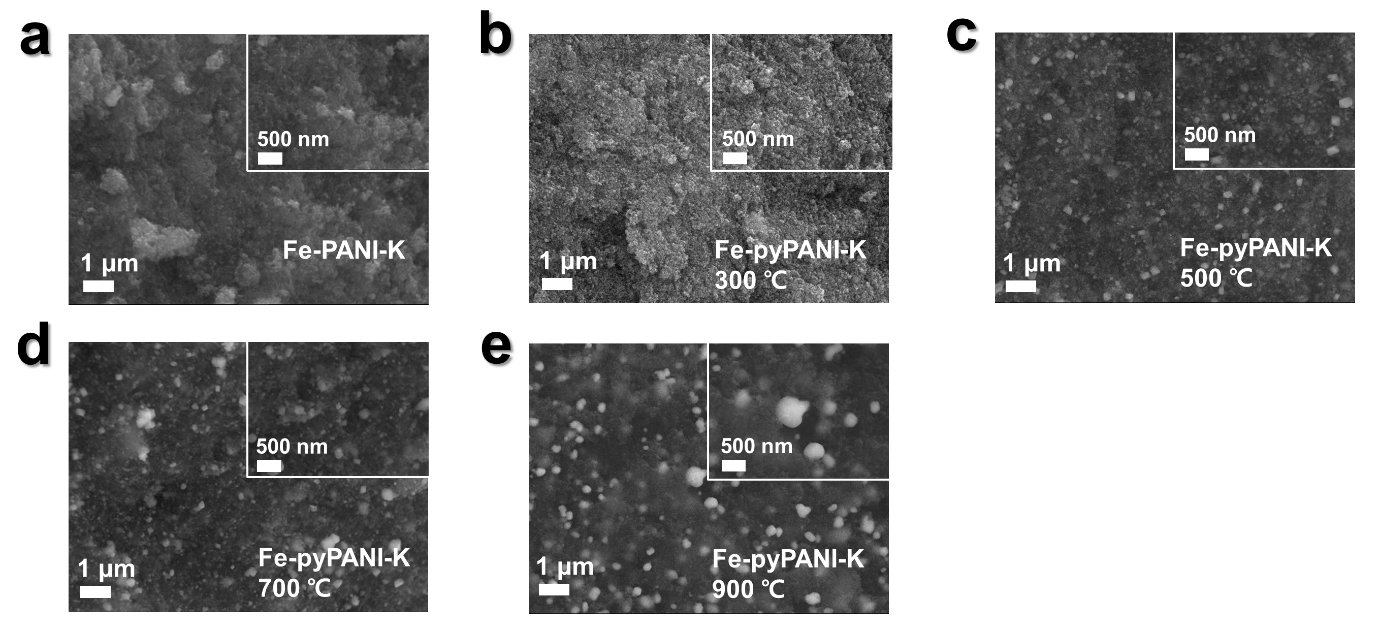


**Figure S2.** Surface FE-SEM images of (**a**) as prepared Fe-PANI-K and Fe-pyPANI-Ks pyrolyzed (**b**) at 300 °C, (**c**) at 500 °C, (**d**) 700 °C, and (**e**) 900 °C.


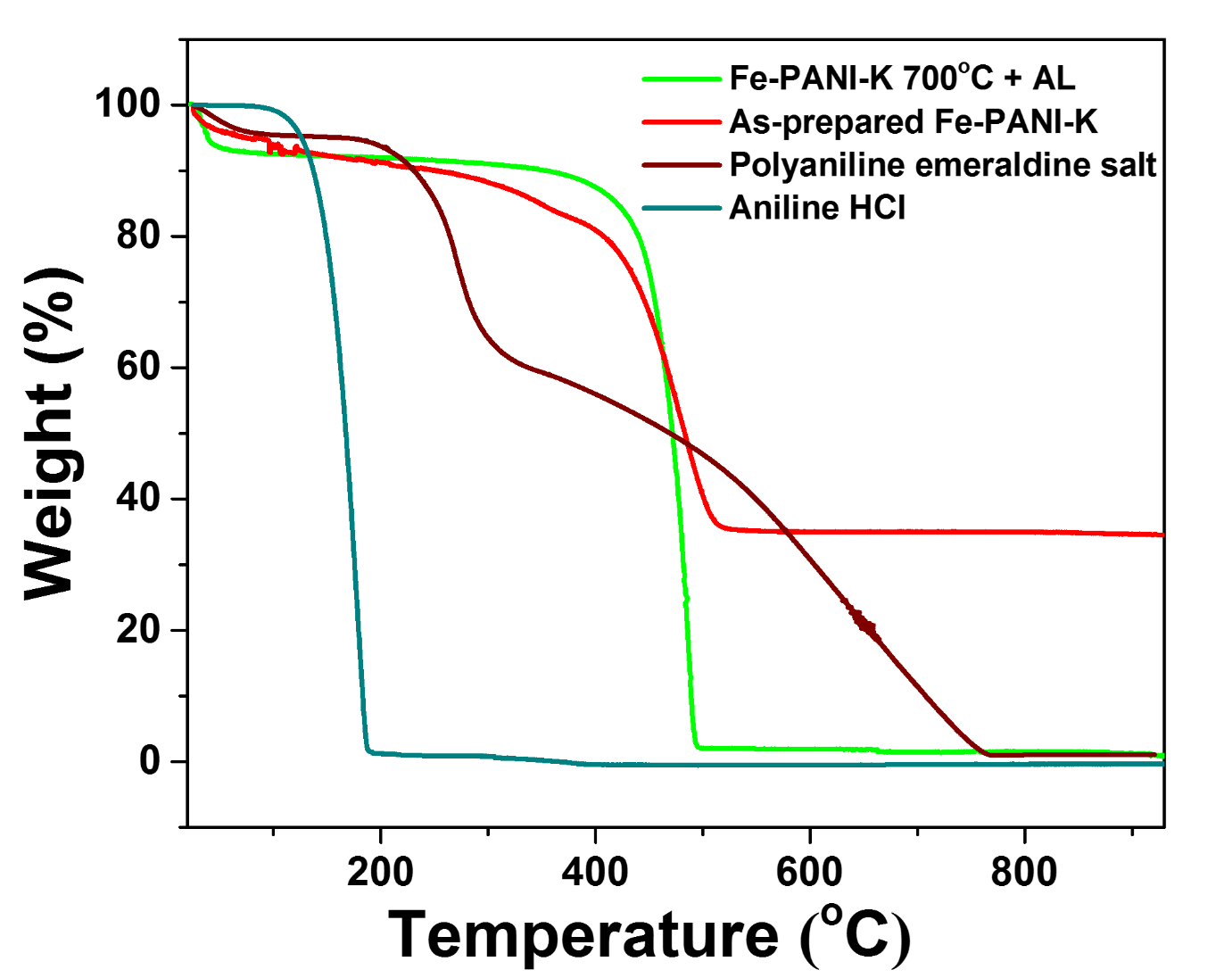


**Figure S3.** TGA analysis of aniline monomer, commercial polyaniline emeraldine salt (MW 15,000) and Fe-PANI-Ks.


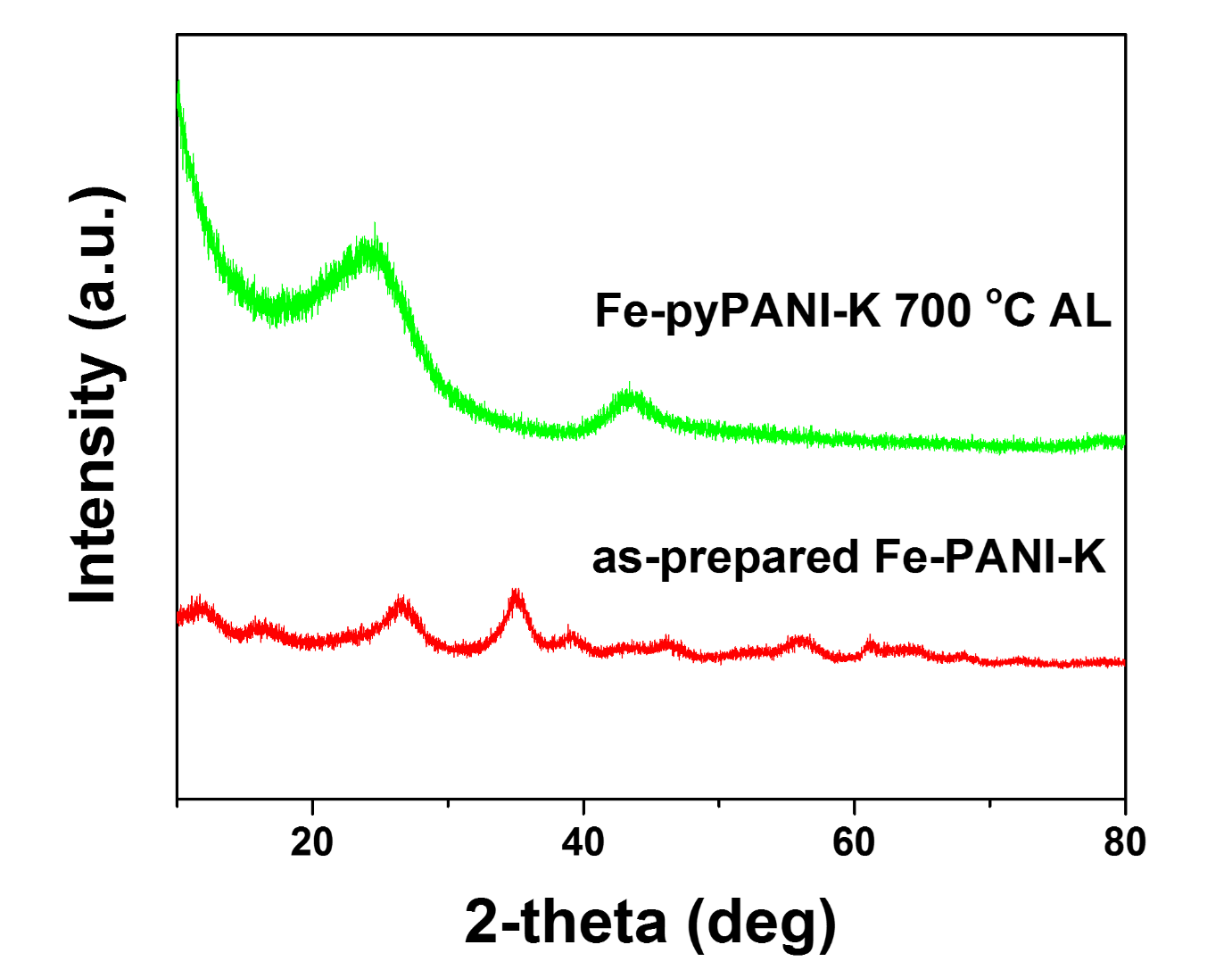


**Figure S4.** XRD patterns of as-prepared Fe-PANI-K and Fe-PANI-K 700 °C AL.


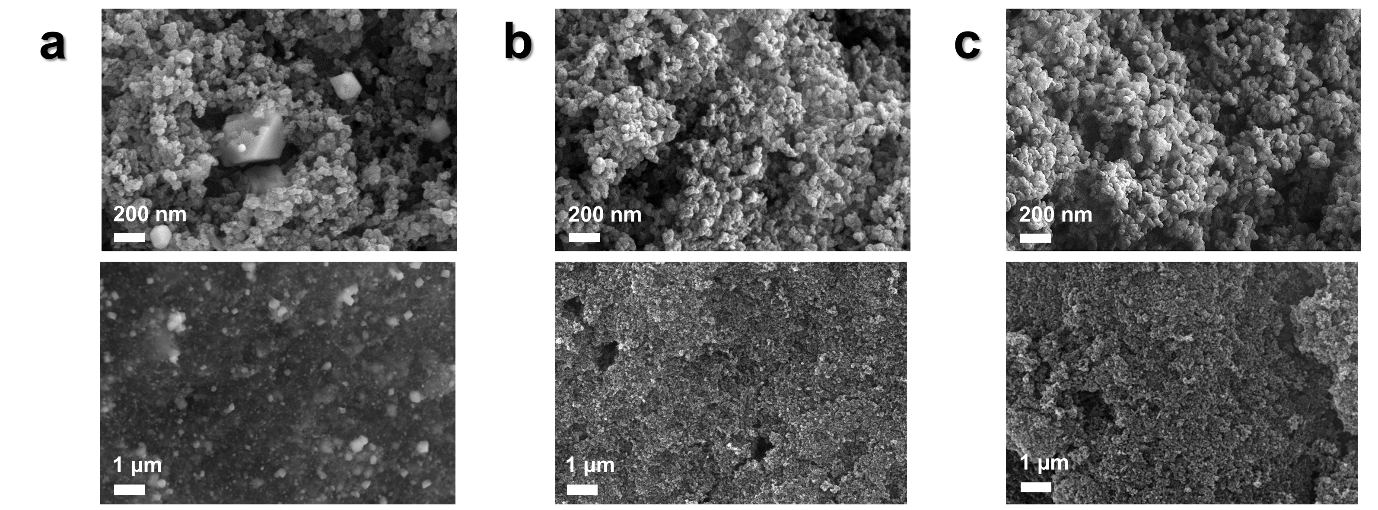


**Figure S5.** Surface FE-SEM images of (**a**) Fe-pyPANI-K 700 °C, (**b**) Fe-pyPANI-K 700 °C AL, (**c**) Fe-pyPANI-K 700 °C after second heat treatment.

**
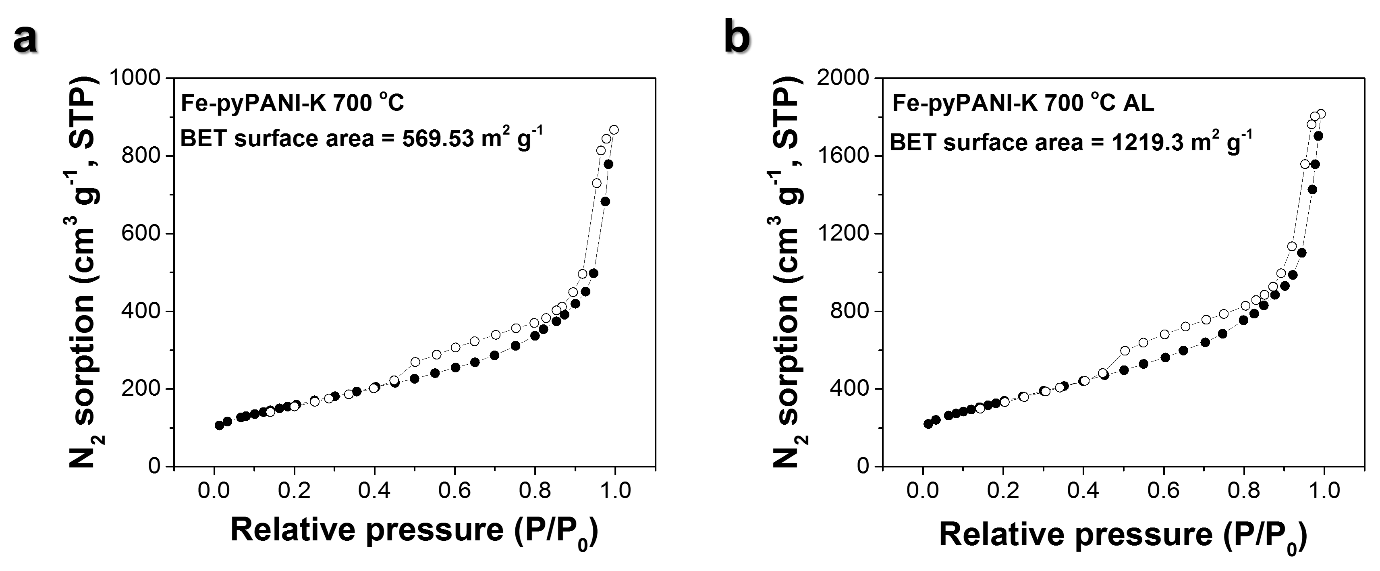
**

**Figure S6.** N_2_ adsorption-desorption isotherm of (**a**) Fe-pyPANI-K 700 °C and (**b**) Fe-pyPANI-K 700 °C AL.


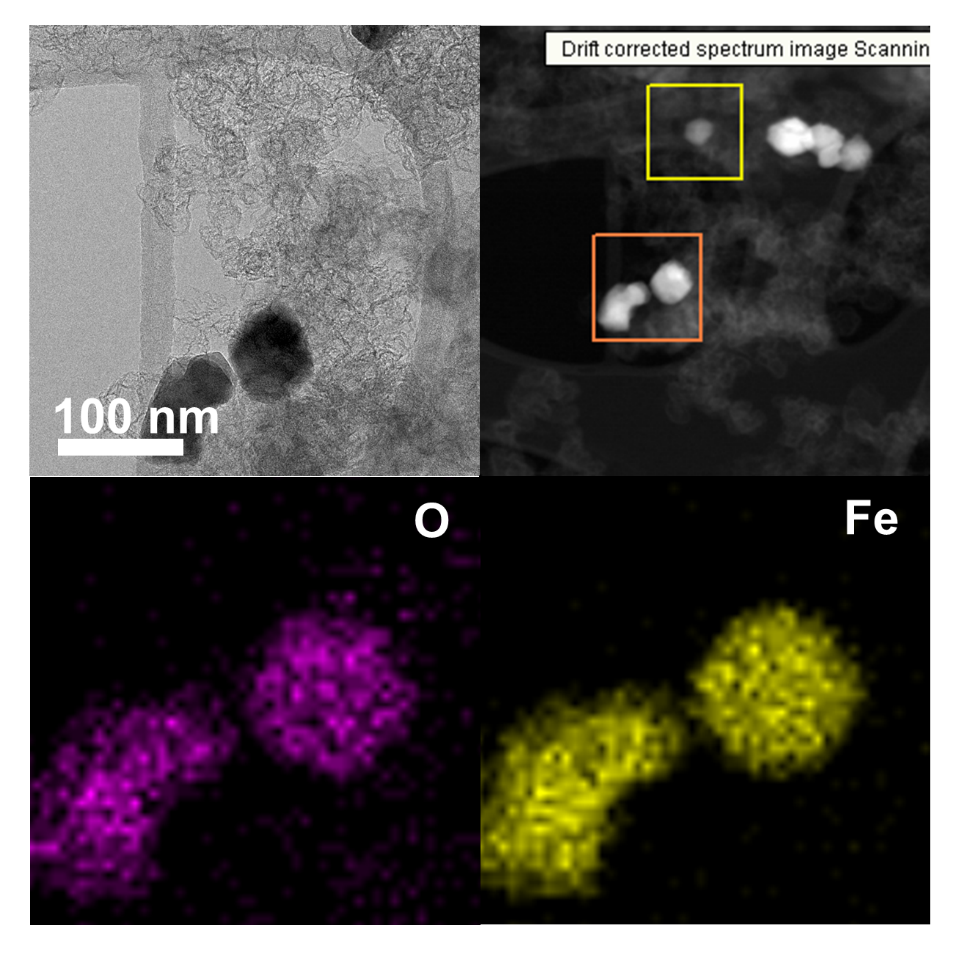


**Figure S7.** EDS mapping image of Fe-pyPANI-K 700 °C.


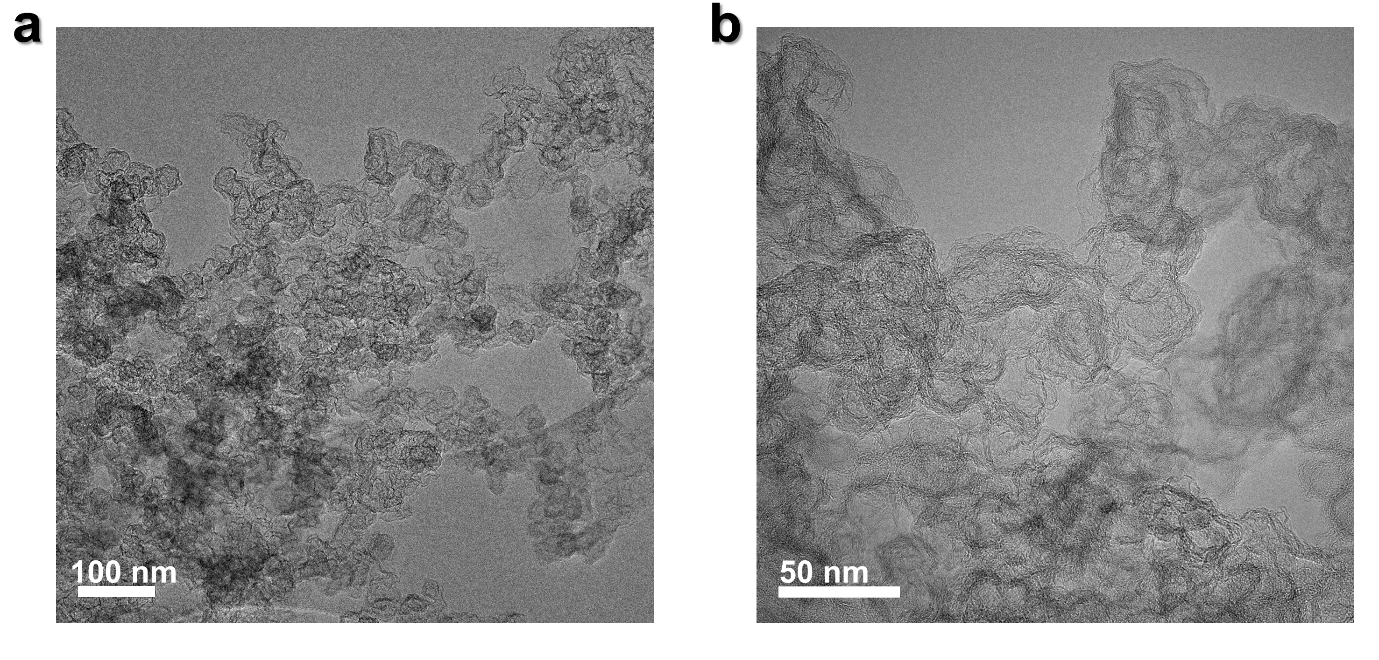


**Figure S8**. TEM images of carbon black. (AkzoNobel, Ketjen black EC-300J)


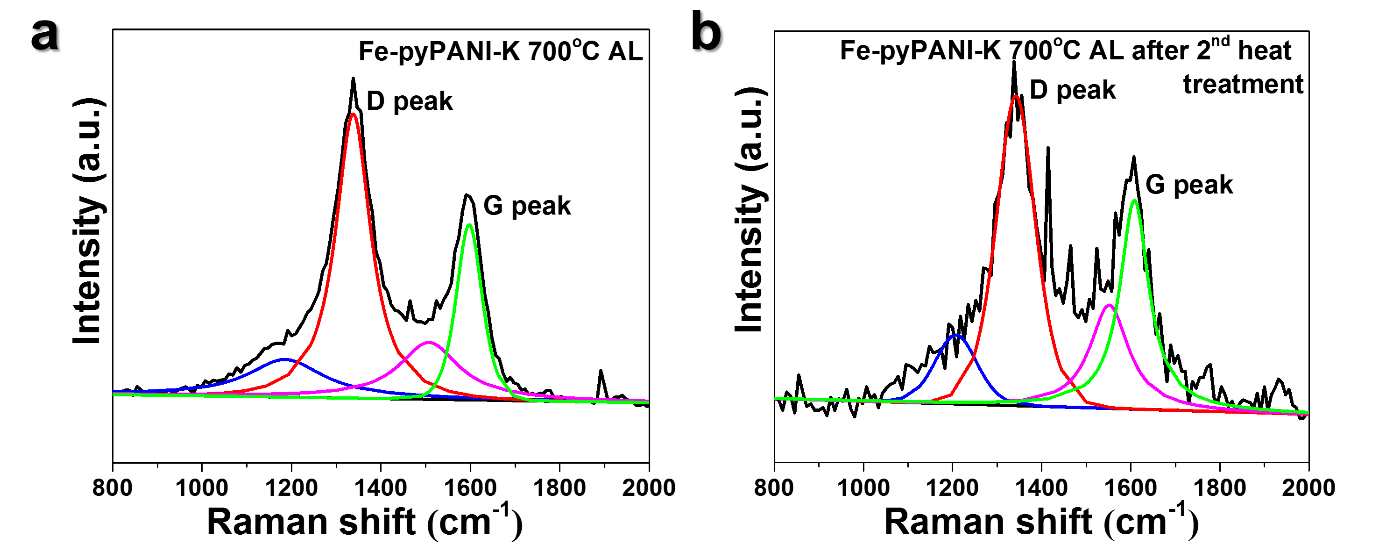


**Figure S9.** Raman spectra of Fe-pyPANI-K 700 °C (**a**) after acid leaching process and (**b**) following second heat treatment.


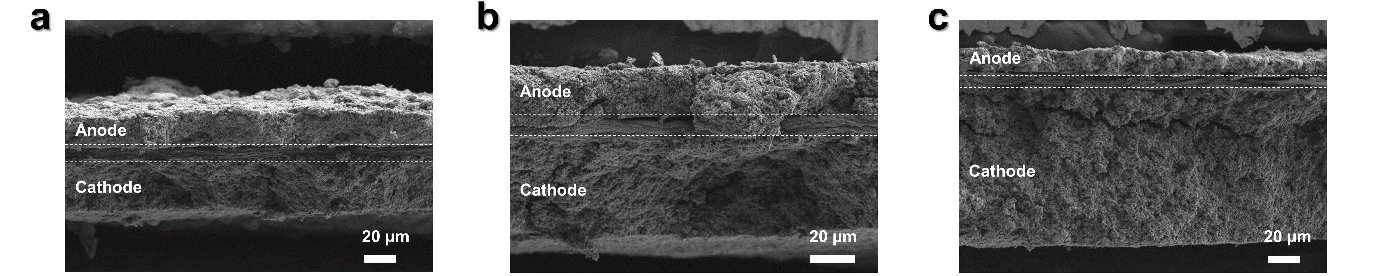


**Figure S10.** Cross-sectional analysis of MEAs with different Fe-pyPANI-K 700 °C loading (**a**) 1 mg cm^-2^ (**b**) 2 mg cm^-2^ (**c**) 3 mg cm^-2^.
